# Introduction to the Peer Teacher Training in health professional education supplement series

**DOI:** 10.1186/s12909-020-02279-y

**Published:** 2020-12-03

**Authors:** Annette Burgess, Christie van Diggele, Chris Roberts, Craig Mellis

**Affiliations:** 1 The University of Sydney, Faculty of Medicine and Health, Sydney Medical School, Education Office, The University of Sydney, Edward Ford Building A27, Sydney, NSW 2006 Australia; 2 The University of Sydney, Faculty of Medicine and Health, Sydney Health Professional Education Research Network, The University of Sydney, Sydney, Australia; 3 The University of Sydney, Faculty of Medicine and Health, The University of Sydney, Sydney, Australia; 4 The University of Sydney, Faculty of Medicine and Health, Sydney Medical School, Central Clinical School, The University of Sydney, Sydney, Australia

**Keywords:** Peer teacher training, Clinical teacher training, Interprofessional, Feedback, Professional skills

## Abstract

Skills in supervision, teaching, facilitation, assessment and feedback, leadership and interprofessional teamwork are required graduate attributes for health professionals. Despite this, the opportunity for learning these skills is rarely embedded within undergraduate and postgraduate health professional training curricula. Additionally, there are limited examples of interprofessional delivery of teaching programs. Since teaching skills can be learned, healthcare faculties play an important role in improving the teaching abilities of their students. At the University of Sydney, we developed and implemented interprofessional, blended learning teacher training programs for health professional students, and junior health professionals: The Peer Teacher Training (PTT) program, and the Clinical Teacher Training (CTT) program. Based on our successful programs, this paper provides an introduction to our Peer Teacher Training supplement. Namely, 11 articles designed to assist those who work and teach in a clinical context; address key challenges; and provide practical tips and frameworks to assist in teaching, assessment, and feedback.

## Background

Teaching is a core professional skill required by all health professionals, from new graduates to experienced clinicians, and academics. Health professionals are not only expected to teach their peers and juniors within their own disciplines, but also across a range of health disciplines. Skills in supervision, teaching, facilitation, assessment and feedback, leadership and interprofessional teamwork are required graduate attributes for health professionals [1–3]. Teacher training programs are not only necessary to prepare health professional students for future practice in the workforce, but also for participation in peer assisted learning (PAL) activities that support university curricula. The need for professional development in these areas for healthcare students and junior healthcare practitioners is widely acknowledged. Despite this, the opportunity for learning these skills is rarely embedded within undergraduate and postgraduate health professional training curricula.

### What is peer assisted learning (PAL)?

Peer assisted learning (PAL) is described as *“people from similar social groupings who are not professional teachers helping each other to learn and learning themselves by teaching*” [4]. As a pedagogy, PAL has the capacity to address specific gaps within curricula [5, 6], and provide qualitatively different experiences to traditional teaching by faculty [4]. Although the vertical integration of health professional curricula provides early patient exposure within clinical settings, the availability of clinicians to teach is a well recognised resource issue [7, 8]. This highlights the importance of the provision of additional support, including peer assisted learning. PAL activities, including peer teaching, assessment and feedback, are well accepted as support resources in many health curricula, where participation and learning involve a process of socialisation. Some examples of our published PAL activities at the University of Sydney are provided in Table 1 [9–14].

**Table 1:** Examples of PAL activities at the University of Sydney

● Senior students as simulated patients in practice OSCE [9] ● Senior students as examiners of their junior peers in practice OSCE [10] ● Senior students as co-examiners of their peers (alongside academic co-examiners) in formative clinical long case examinations [11–13] ● Senior students as peer tutors in the clinical setting [14]	

Although reports of formal and informal peer assisted learning activities are aplenty, there is paucity of reports on preparation and training requirements [15, 16]. For example, a recent systematic review of teacher training programs within the discipline of medicine, found variations in the necessary preparation for peer teaching activities, with little or no assessment of competence prior to participation [15]. However, teaching skills are best acquired through training, opportunities for practice, and provision of specific feedback [16].

### Development of the Peer Teacher Training (PTT) program

In 2016, we developed an up to date, innovative *“Peer Teacher Training”* (PTT) program for health professional students [17], based on best practice, and consisting of six modules. This PTT program has been previously described [17]. In 2017, we built on the existing PTT program to develop an eight module *“Clinical Teacher Training”* (CTT) program targeting junior health professionals [18]. These two programs adopted both an interprofessional and a flipped classroom approach. We had two broad aims; first to promote engagement in the development of learning and teaching, assessment and feedback skills; second, to promote engagement with interprofessional education.

### The aim of the Peer Teacher Training supplement

The aim of this “Peer Teacher Training in health professional education supplement” is to provide theoretical background, insight and tips for health professional students and junior health professionals, based on the experience and implementation of our teacher training programs. Since 2016, our PTT and CTT programs have been used to train over 1000 senior health professional students and staff (including pharmacy, allied health, nursing, dentistry, and medicine). This Supplement is designed to assist those who work and teach in a clinical context, address key challenges and provide practical tips and frameworks to assist in teaching, assessment, and feedback. Although each article is designed to be read individually, there is continuity of themes throughout the Supplement. The title of each article is listed in Table 2, and each article provides theoretical background, practical examples and activities. They are designed as a platform for busy health professional students and junior clinicians to work towards developing skills in communication, teaching, assessment and feedback.

**Table 2:** Articles in the Peer Teacher Training in health professional education supplement

1. Introduction to the Peer Teacher Training in health professional education supplement series	
2. Feedback in the clinical setting	
3. Planning, preparing and structuring a small group teaching session	
4. Facilitating small group learning in the health professions	
5. Key tips for teaching in the clinical setting	
6. Tips for teaching procedural skills	
7. Teaching clinical handover with ISBAR	
8. Interprofessional Education: tips for design and implementation	
9. Team-based learning: design, facilitation and participation	
10. Leadership in health professional education	
11. Planning Peer Assisted Learning (PAL) activities in clinical schools	

### Why the emphasis on interprofessionalism?

The World Health Organisation (WHO) has identified that effective collaboration between health professionals plays an important role in preparing and providing the health workforce with the ability to respond to local health needs and provide strengthened health systems [19]. Implementation of university health education learning activities within an interprofessional context has the potential to improve patient safety through improvements in leadership skills, collaboration, and communication between healthcare teams [20–22]. However, limited examples of structured interprofessional learning (IPL) activities have been identified within university health professional curricula [23, 24]. There are obvious barriers to the delivery of interprofessional learning activities, such as timetable restrictions, cultural barriers within organisations, negative attitudes, and the preferred isolation of disciplines (Table 3) [25–27]. We have written these articles and related activities within an interprofessional context, to encourage collaboration between health professional students. We found interprofessionalism to be the most valued feature of both the PTT and CTT programs [17, 18]. Our students have embraced the rare opportunity for formal collaboration in meaningful activities. Further, faculty, from across disciplines, have enjoyed working together in delivering the programs. We now have a large group of alumni who assist in facilitation of the PTT and CTT programs, and the programs are run across a number of hospital and university campuses.

**Table 3:** Barriers and enablers of IPL

Potential barriers to IPL	Potential enablers of IPL
● Timetabling clashes and restrictions	● Embedding interprofessional activities within curricula
● Cultural barriers within organisations and departments	● IPL ‘champions’ among disciplines
● Preference of disciplines to work in their silos	● Leadership in IPL from management
● Negative attitudes of faculty and departments	● Enthusiasm towards IPL within organisations
● Students’ negative attitudes towards professions	● Adequate knowledge and understanding of other professions
● Inadequate preparation by participants	● Adequate preparation of learners prior to IPL activities

### Qualities of effective clinical teachers

Many of the attributes associated with excellence in role modelling within the healthcare professions relate to teaching skills [28, 29]. Teaching abilities, dedication to teaching, and an ability to facilitate students’ learning needs through rapport, encouragement, and constructive feedback, are all elements that contribute. The extent to which learners are able to engage and learn from clinical tutorials relies on multiple elements, including the planning and structure of tutorials, the attitude of both the teacher and learners, and their mutual understanding of learning outcomes [30]. All learners, including adult learners, have different learning style preferences, and it is important to ensure that teaching styles are flexible enough to accommodate a range of learners. Examples of qualities of an effective teacher are summarised in Table 4 [31].

**Table 4:** Qualities of effective teachers (adapted from Rose & Best, 2005) [31]

Quality	Characteristic
Organisation and clarity	Explains clearly Presents material in an organised way Summarises and emphasises what is important Communicates what is expected to be learnt
Group instruction skills	Establishes rapport with students Shows respect for and interest in students
Enthusiasm	Is dynamic and energetic Enjoys teaching Stimulates interest/curiosity in the subject
Knowledge	Is up-to-date with current practice and related research Discusses divergent points of view
Clinical supervision	Demonstrates clinical procedures Provides practice opportunities Offers professional support and encouragement Observes student performance Identifies strengths and limitations objectively Provides feedback and positive reinforcement
Clinical competence	Demonstrates skill in synthesising and managing patient problems Maintains a holistic orientation with patients Works effectively within a healthcare team

## Conclusion

Since teaching skills can be learned, healthcare faculties play an important role in improving the teaching abilities of their students. Development of effective clinical teachers, equipped with optimal teaching strategies should be supported during university education and beyond. This means utilising the best available evidence regarding methods and tools to assist in this endeavour. For busy students, staff and faculty teachers, the success of our PTT and CTT programs [17, 18] serves as a model for both “interprofessional” and “flipped” learning designs. Our programs were specifically designed to develop students’ knowledge and skills through pre-class preparation, followed by face-to-face class teaching which included; small group teaching, interprofessional activities, and formative assessment with feedback. This Supplement is intended to provide an educational framework upon which students and junior clinicians can build on their competence. We have included instructions in general elements of teaching, and at the same time, provided detailed theory and references for those who wish to explore areas in greater depth.

## Data Availability

Not applicable.

## Abbreviations

PTT: Peer Teacher Training
CTT: Clinical Teacher Training
PAL: Peer Assisted Learning
IPL: Interprofessional learning
OSCE: Objective Structured Clinical Examination
ISBAR: Introduction, Situation, Background, Assessment, Recommendation
WHO: The World Health Organisation
